# Supplementary material for: Solid Lipid Nanoparticles Based on Monosubstituted Pillar[5]arenes: Chemoselective Synthesis of Macrocycles and Their Supramolecular Self-Assembly
Source: Nanomaterials (Basel). 2022 Nov 30;12(23):4266. doi: 10.3390/nano12234266 (PMC9738619; doi:10.3390/nano12234266)
Supplement: Supplementary file 1 [file nanomaterials-12-04266-s001.zip › nanomaterials-2049913-supplementary.pdf]

Supplementary Materials

Solid Lipid Nanoparticles Based on Monosubstituted Pillar[5]arenes: Chemoselective Synthesis of Macrocycles and Their Supramolecular Self-Assembly

Darya Filimonova [1], Anastasia Nazarova [1,*], Luidmila Yakimova [1,*] and Ivan Stoikov [1,2]

[1] A.M. Butlerov Chemistry Institute of Kazan Federal University, 18 Kremlyovskaya Str., 420008 Kazan, Russia
[2] Federal State Budgetary Scientific Institution «Federal Center for toxicological, radiation, and biological safety», Nauchny Gorodok-2, 420075 Kazan, Russia
* Correspondence: anas7tasia@gmail.com (A.N.); mila.yakimova@mail.ru (L.Y.); Tel.: +7-843-233-7241

1. Synthesis

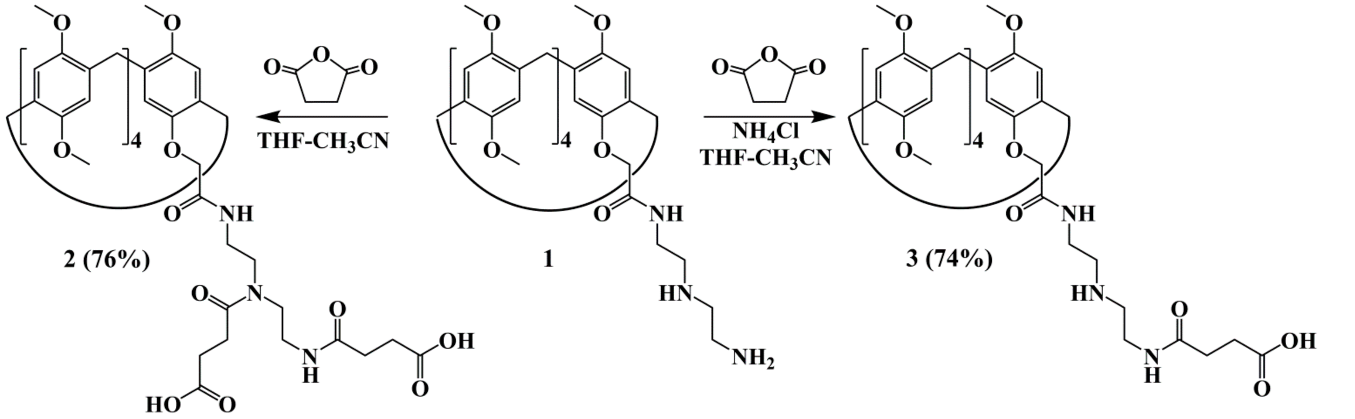

2. Materials and Methods

2.1. General

^{1}H NMR, ^{13}C and 2D NOESY NMR spectra were obtained on a Bruker Avance-400 spectrometer (Bruker Corp., Billerica, MA, USA) (^{13}C{1H} – 100 MHz and ^{1}H and 2D NOESY – 400 MHz). The chemical shifts were determined against the signals of residual protons of deuterated solvent ($CDCl_3$, DMSO-d_6). The concentrations of the compounds were equal to 3–5% by the weight in all the records. The FTIR ATR spectra were recorded on the Spectrum 400 FT-IR spectrometer (Perkin Elmer Inc, Waltham, MA, USA) with a Diamond KRS-5 attenuated total internal reflectance attachment (resolution 0.5 cm^{-1}, accumulation of 64 scans, recording time 16 s in the wavelength range 400–4000 cm^{-1}). Mass spectra were obtained on a Bruker Ultraflex III MALDI-TOF instrument (Bruker Daltonik GmbH, Bremen, Germany) with *p*-nitroaniline as the matrix. Elemental analysis was performed on Perkin–Elmer 2400 Series II instruments (Perkin Elmer, Waltham, MA, USA). Melting points were determined using Boetius Block apparatus (VEB Kombinat Nagema, Radebeul, Germany).

Succinic anhydride and ammonium chloride were purchased from Acros (USA) and used as received. Chemically pure organic solvents were purified by standard methods.

All the aqueous solutions were prepared with the Millipore-Q deionized water (>18.0 MW cm at 298 K).

Pillar[5]arene **1** was synthesized according to the literature [S1].

4-((2-(3-Carboxy-N-(2-(2-acetamido)ethyl)propaneamido)ethyl)amino)–8,14,18,23,26,28,31,32,35-nonamethoxy–pillar[5]arene (**2**). Macrocycle **1** (0.20 g, 0.20 mmol) was dissolved in the mixture of 6 mL of tetrahydrofuran and 2 mL of acetonitrile in a round-bottom flask equipped with a magnetic stirrer. Then, 0.09 g (0.90 mmol) of succinic anhydride was added. The reaction mixture was refluxed for 20 hrs. The solvent was removed under reduced pressure at the end of the synthesis. The product was Yield 0.152 g (76%). D.P. = 104 °C. ^{1}H NMR (400 MHz, 298 K, DMSO-*d*$_6$): δ 2.26-2.56 (m, 8H, C(O)CH_2CH_2), 3.14-3.29 (m, 8H, NCH_2CH_2NH), 3.47-3.62 (m, 37H, ArCH_2Ar and ArOCH_3), 4.30 (s, 1H, ArOCH_2), 4.34 (s, 1H, ArOCH_2), 6.70-6.83 (m, 10H, ArH), 7.92 (t, $^3J_{HH}$ = 5.6 Hz, 0.5H, CH_2NHC(O)), 8.06 (t, $^3J_{HH}$ = 5.4 Hz, 0.5H, CH_2NHC(O)), 8.19 (t, $^3J_{HH}$ = 5.6 Hz, 0.5H, C(O)NHCH_2), 8.32 (t, $^3J_{HH}$ = 5.8 Hz, 0.5H, C(O)NHCH_2). ^{13}C NMR (100 MHz, $CDCl_3$): δ 31.6, 32.3, 32.4, 32.6, 33.9, 38.0, 47.0, 56.8, 68.1, 114.6, 115.6, 117.6, 132.5, 133.2, 133.6, 150.2, 150.9, 151.3, 169.4, 172.5, 176.6, 177.8. IR, ν/cm^{-1}: 1540, 1634 (C=O), 1723, 2828, 2852, 2935 (COOH), 3319 (NH). MS (MALDI-TOF) calc. [M+] *m/z* = 1079.5, found $[M+H]^+$ *m/z* = 1080.2, $[M+Na]^+$ *m/z* = 1103.3, $[M+K]^+$ *m/z* = 1119.3. Found (%): C, 64.87; H, 6.38; N, 3.85. Calc. for $C_{58}H_{69}N_3O_{17}$. (%): C, 64.90; H, 6.44; N, 3.89.

4-((2-Aminoethyl)(2-(3-carboxypropaneamido)ethyl)amino)–8,14,18,23,26,28,31,32,35-nonamethoxy-pillar[5]arene (**3**). Pillar[5]arene **1** (0.1 g, 0.1 mmol) was dissolved in the mixture of 6 mL of tetrahydrofuran and 2 mL of acetonitrile in a round-bottom flask equipped with a magnetic stirrer. Ammonium chloride (0.02 g, 0.3 mmol) was preliminarily dissolved in 0.5 mL of water and then added to the reaction mixture. Then, 0.04 g (0.4 mmol) of succinic anhydride was added. The reaction mixture was refluxed for 20 hours. The solvent was removed under reduced pressure at the end of the synthesis. The product was recrystallized from water.

Yield 0.082 g (74%). D.P. = 120°C. ^{1}H NMR (400 MHz, 298 K, DMSO-*d*$_6$): δ 2.28 (AB part of AA’BB’ system, $^3J_{HAHB}$ = 6.4 Hz, $^2J_{HAHA'}$ = 12.8 Hz, 2H, C(O)CH_2CH_2), 2.37 (A’B’ part AA’BB’ system, $^3J_{HAHB}$ = 6.4 Hz, $^2J_{HAHA'}$ = 12.8 Hz, 2H, C(O)CH_2CH_2), 2.43-2.46 (m, 4H, NHCH_2CH_2NHC(O)), 2.98-3.06 (m, 4H, C(O)NHCH_2CH_2NH), 3.64-3.66 (m, 37H, ArCH_2Ar and ArOCH_3), 4.38 (s, 2H, ArOCH_2), 6.72-6.84 (m, 10H, ArH), 8.19 (t, $^3J_{HH}$ = 5.6 Hz, 1H, CH_2NHC(O)), 8.36 (t, $^3J_{HH}$ = 6.0 Hz, 1H, C(O)NHCH_2,), 8.72-8.86 (m, 1H, CH_2NHCH_2). ^{13}C NMR (100 MHz, $CDCl_3$): δ 29.8, 32.4, 32.6, 33.9, 38.8, 50.0, 55.9, 56.8, 113.0, 113.6, 114.2, 116.6, 128.3, 131.5, 132.2, 132.6, 149.2, 149.9, 150.6, 169.7, 174.6. IR, ν/cm^{-1}: 1205 (COOH), 1654 (C=O), 1703, 2933 (COOH), 3333 (NH). MS (MALDI-TOF) calc. [M+] *m/z* = 979.4, found $[M+H]^+$ *m/z* = 980.6. Found (%): C, 66.24; H, 6.63; N, 4.30. Calc. for $C_{54}H_{65}N_3O_{14}$. (%): C, 66.17; H, 6.68; N, 4.29.

2.2. SLN Preparation

Monosubstituted pillar[5]arene **2** (**3**) (3 mg) was dissolved in 1 mL of tetrahydrofuran. Ultrapure water (10 mL) was added after 5 minutes stirring. Then, the suspension was dispersed with ultrasound for 15 minutes, after which THF was evaporated at 40 °C under reduced pressure. The concentration of the obtained particles based on the macrocycles **2** and **3** was 3 mg/mL.

2.3. Dynamic Light Scattering (DLS)

2.3.1. Particles’ Size

The distribution of particles by number, volume, and intensity, the polydispersity index were determined by dynamic light scattering (DLS) on a Zetasizer Nano ZS instrument (Malvern Instruments, Worcestershire, UK) in quartz cuvettes. The instrument is equipped with the 4 mW He-Ne laser (633 nm). Measurements were performed at a detection angle of 173°. The error in determining the particle size is less than 2%. The

results were processed by the DTS program (Dispersion Technology Software 4.20). Dimethyl sulfoxide and chloroform were used to prepare solutions for studying the aggregation of pillar[5]arenes **2** and **3**. In the course of the experiment, the concentrations of macrocycles varied within $1 \times 10^{-5} - 1 \times 10^{-3}$ M. Deionized water was used as a solvent to determine the size of the SLN. In the course of the experiment, the concentrations of SLN based on compounds **2** and **3** were varied from 3×10^{-6} to 3×10^{-4} M. The particle sizes were measured after 1 h mixing. Measurements were determined after 24 and 178 h three times to evaluate kinetic stability.

2.4. Electrophoretic Light Scattering (ELS)

Zeta Potentials

Zeta (ζ) potentials were determined by electrophoretic light scattering (ELS) on a Zetasizer Nano ZS from Malvern Instruments (Worcestershire, UK). Samples were prepared as for the DLS measurements and were transferred with the syringe to the disposable folded capillary cell for measurement. The zeta potentials were measured using the Malvern M3-PALS method and averaged from three measurements.

2.5. Transmission electron microscopy (TEM)

TEM measurements were made at the Interdisciplinary Center for Analytical Microscopy of the Kazan Federal University. Analysis of samples was carried out using a Hitachi HT7700 Exalens transmission electron microscope (Tokyo, Japan) with an Oxford Instruments X-Maxn 80T EDS detector working in STEM mode. Samples of SLN-2, and mixed SLN-3 (3×10^{-4} M) were prepared similarly to those studied by the DLS method. 10 μl of the suspension was placed on a carbon-coated 3 mm copper grid and dried at room temperature using special holder for microanalysis. After drying, the grid was placed in the transmission electron microscope and analyzed at an accelerating voltage of 80 kV.

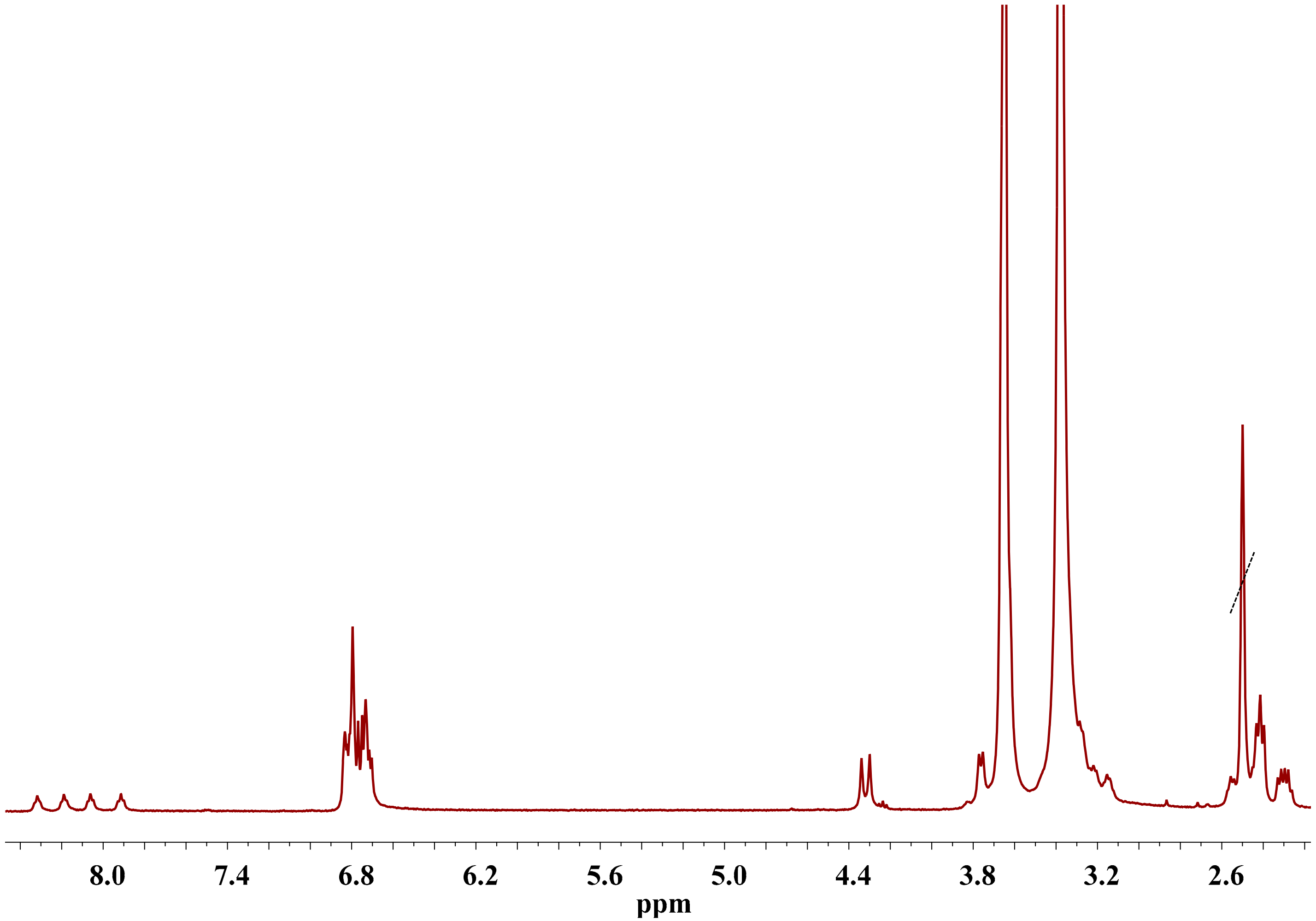

Figure S1. ^{1}H NMR spectrum of 4-((2-(3-carboxy-*N*-(2-(2-acetamido)ethyl)propanamido)ethyl)amino)-4-oxobutanoic acid-8,14,18,23,26,28,31,32,35 -nonamethoxypillar[5]arene (2), DMSO-d_6, 298 K, 400 MHz.

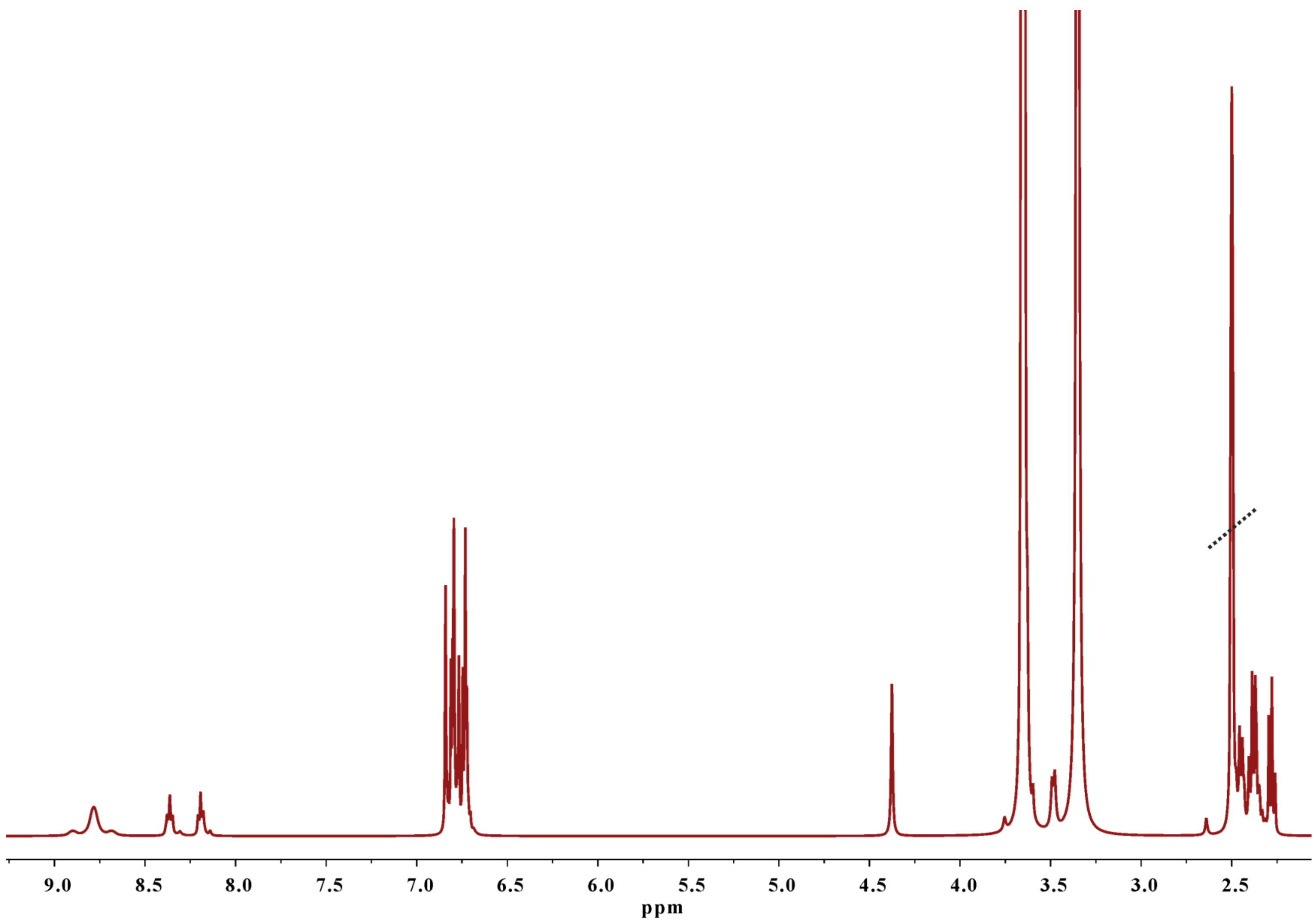

Figure S2. ^1H NMR spectrum of 4-((2-aminoethyl)-(2-(3-carboxypropanamido)ethyl)amino)-4-oxobutanoic acid-8,14,18,23,26,28,31,32,35 -nonamethoxypillar[5]arene (3), DMSO-d_6, 298 K, 400 MHz.

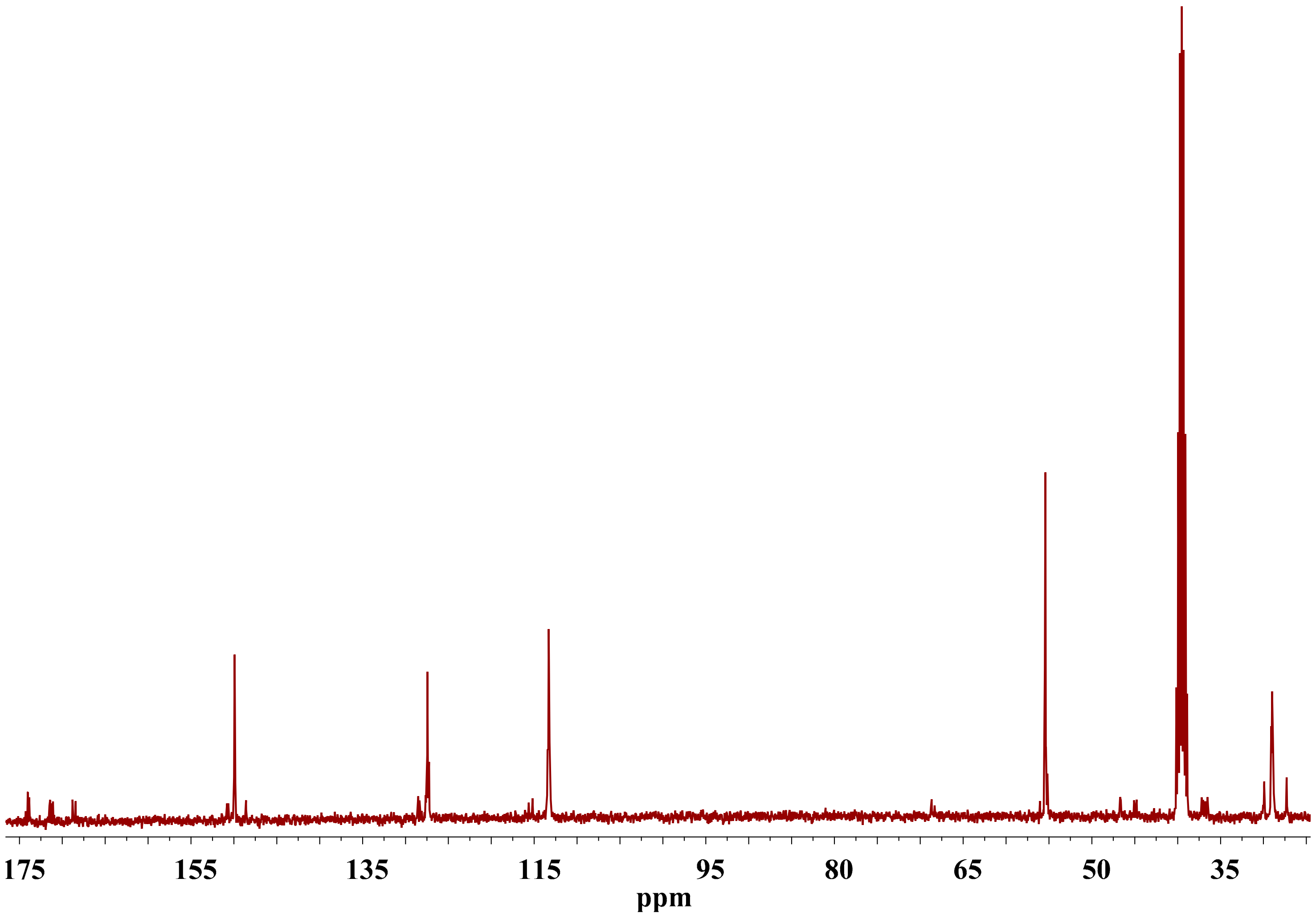

Figure S3. ^{13}C NMR spectrum of 4-((2-(3-carboxy-*N*-(2-(2-acetamido)ethyl)propanamido)ethyl)amino)-4-oxobutanoic acid-8,14,18,23,26,28,31,32,35 -nonamethoxypillar[5]arene (2), DMSO-*d*$_6$, 298 K, 100 MHz.

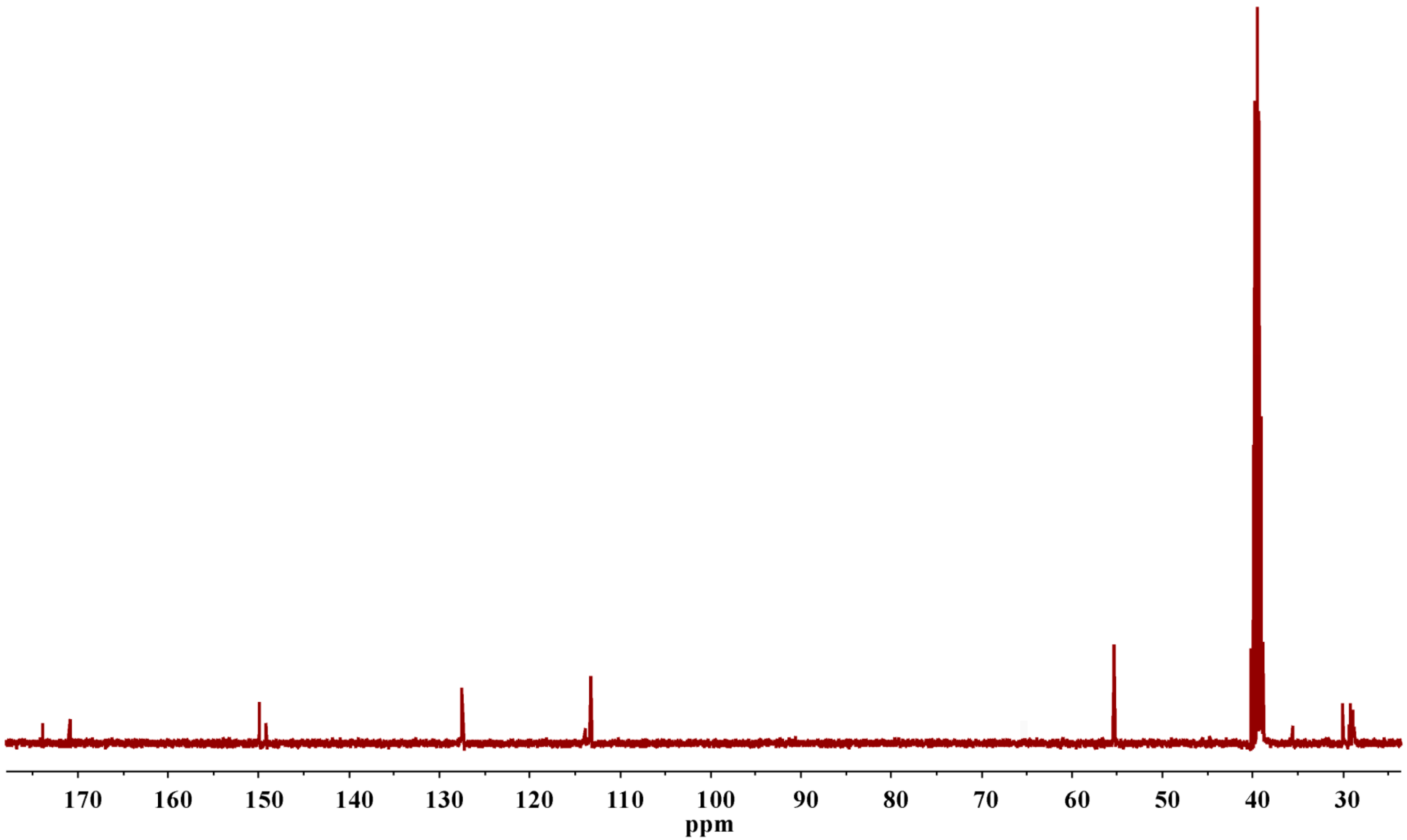

Figure S4. ^{13}C NMR spectrum of 4-((2-aminoethyl)-(2-(3-carboxypropanamido)ethyl)amino)-4-oxobutanoic acid-8,14,18,23,26,28,31,32,35 -nonamethoxypillar[5]arene (4), DMSO-d_6, 298 K, 100 MHz.

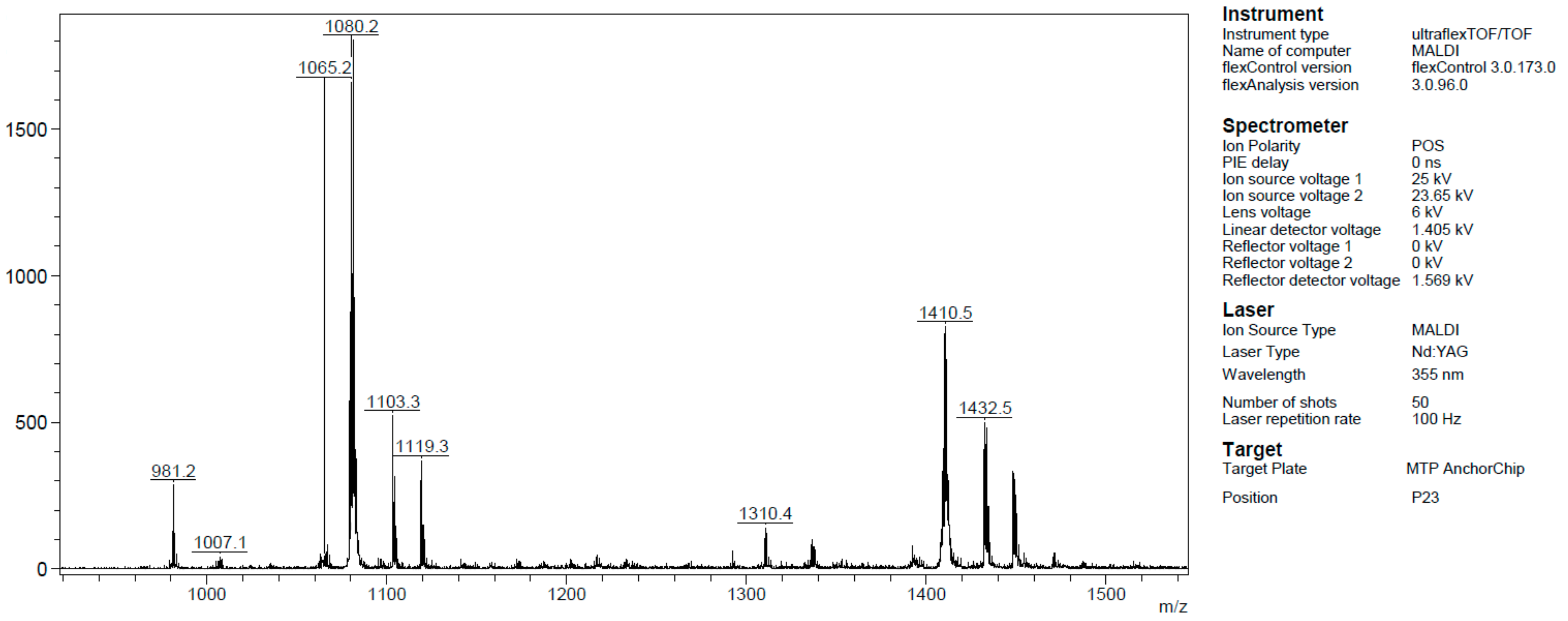

Figure S5. Mass spectrum (MALDI) of 4-((2-(3-carboxy-*N*-(2-(2-acetamido)ethyl)propanamido)ethyl)amino)-4-oxobutanoic acid-8,14,18,23,26,28,31,32,35 - nonamethoxypillar[5]arene (2).

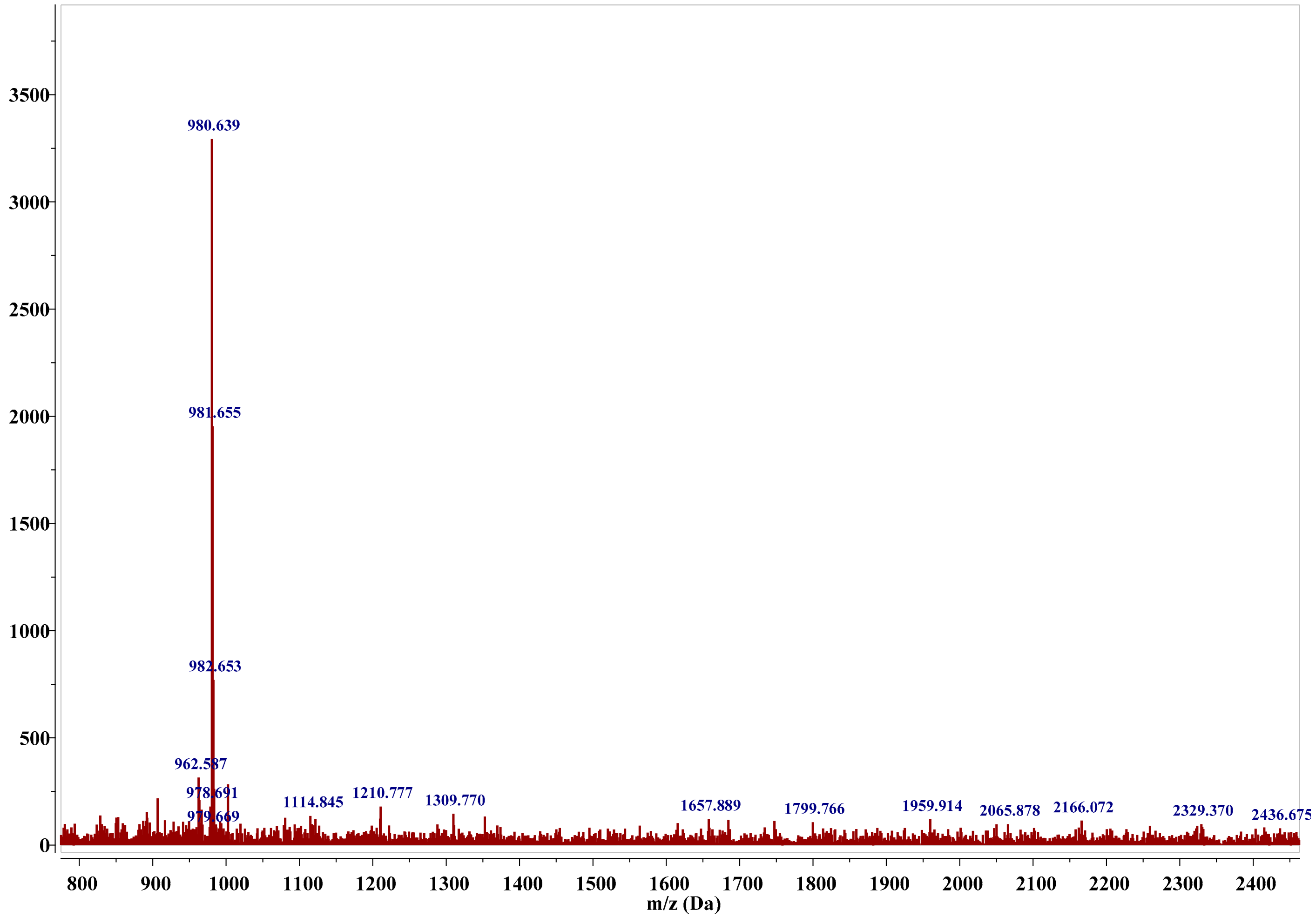

Figure S6. Mass spectrum (MALDI) of 4-((2-aminoethyl)-(2-(3-carboxypropanamido)ethyl)amino)-4-oxobutanoic acid-8,14,18,23,26,28,31,32,35 -nonamethoxypillar[5]arene (3).

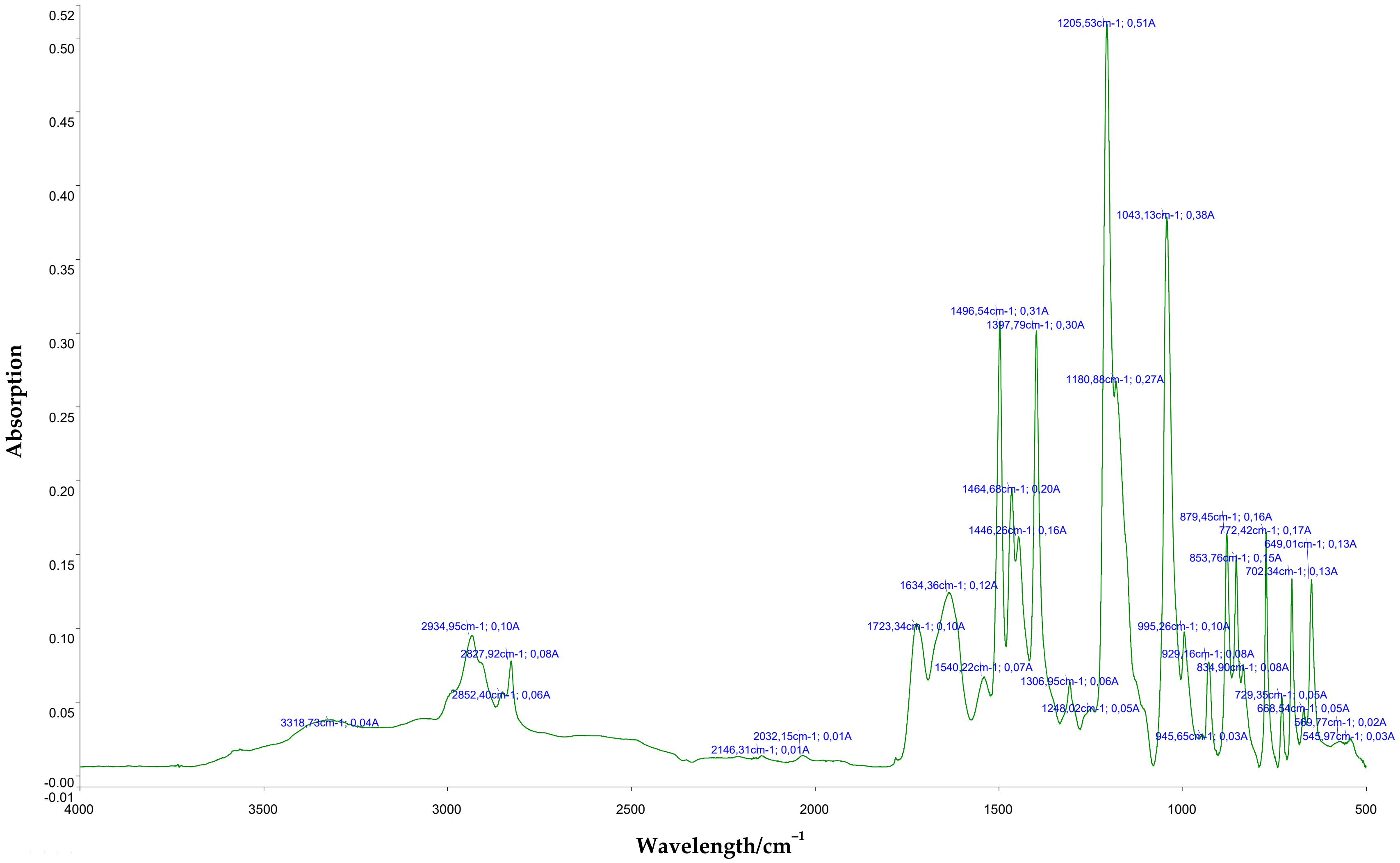

Figure S7. IR spectrum of 4-((2-(3-carboxy-*N*-(2-(2-acetamido)ethyl)propanamido)ethyl)amino)-4-oxobutanoic acid-8,14,18,23,26,28,31,32,35 -nonamethoxypillar[5]arene (2).

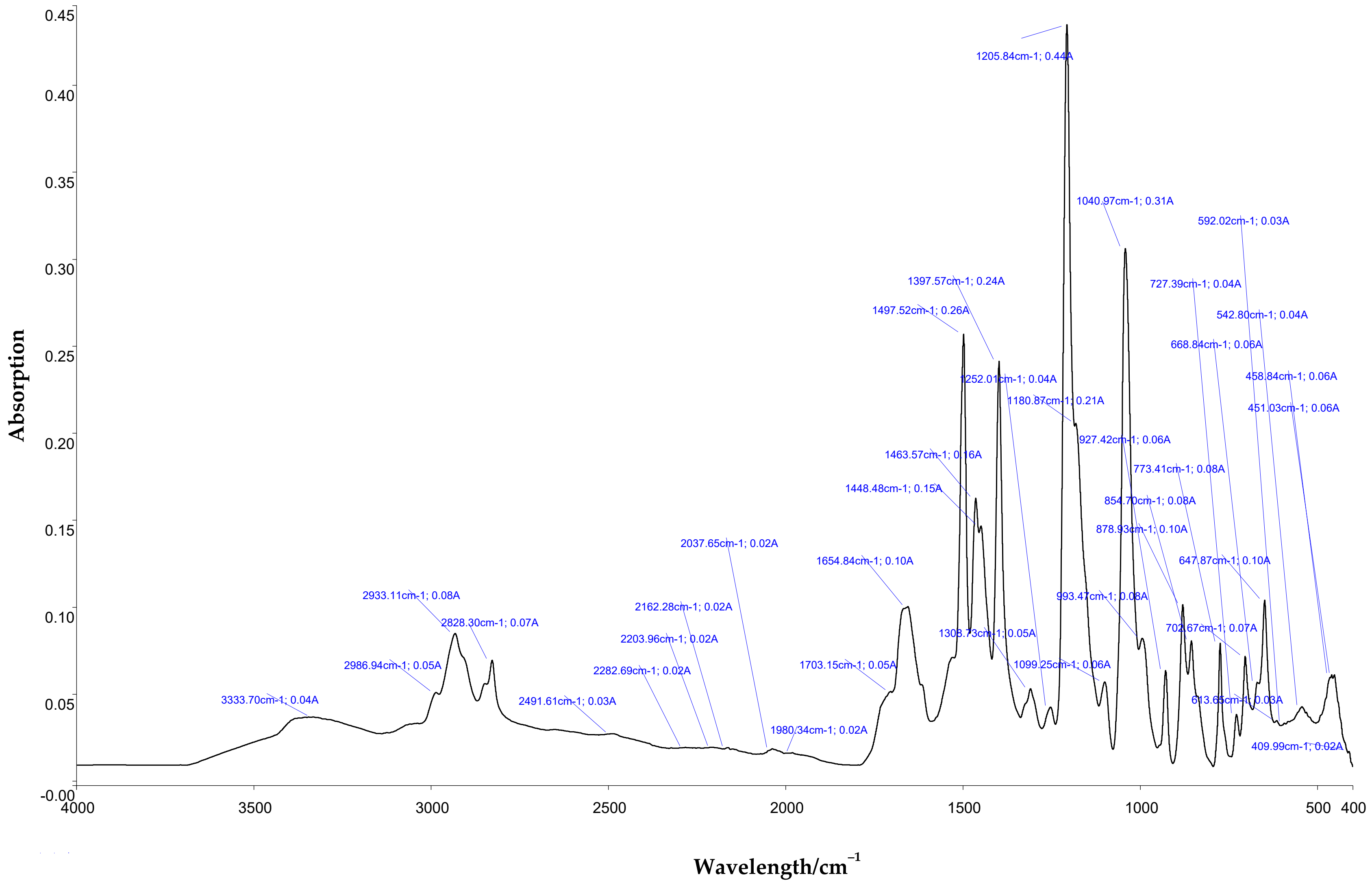

Figure S8. IR spectrum of 4-((2-aminoethyl)-(2-(3-carboxypropanamido)ethyl)amino)-4-oxobutanoic acid-8,14,18,23,26,28,31,32,35 - nonamethoxy-pillar[5]arene (3).

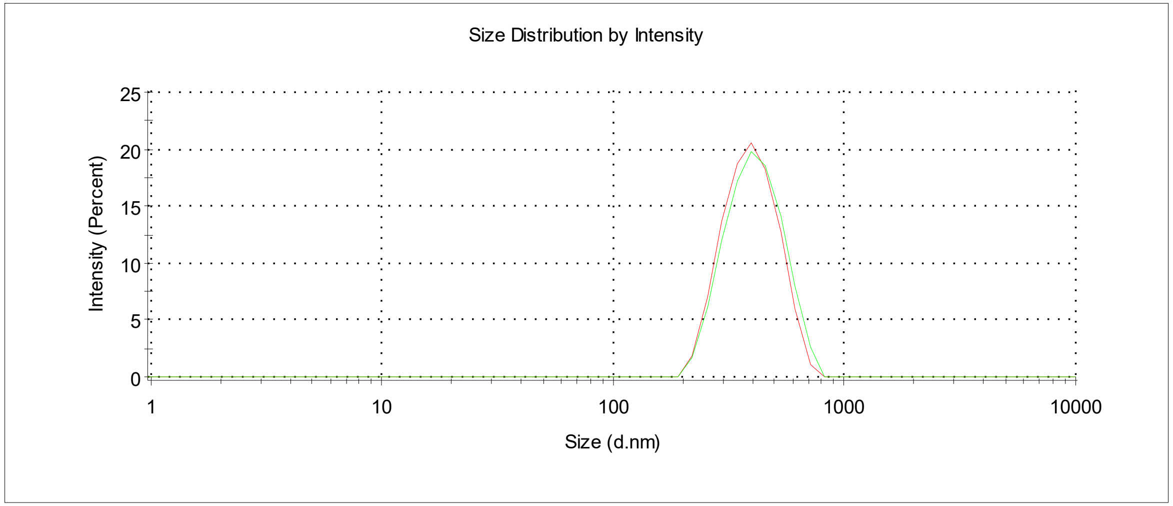

Figure S9. Size distribution of particles by intensity for macrocycle 2 ($1{\times}10^{-4}$ M) in DMSO.

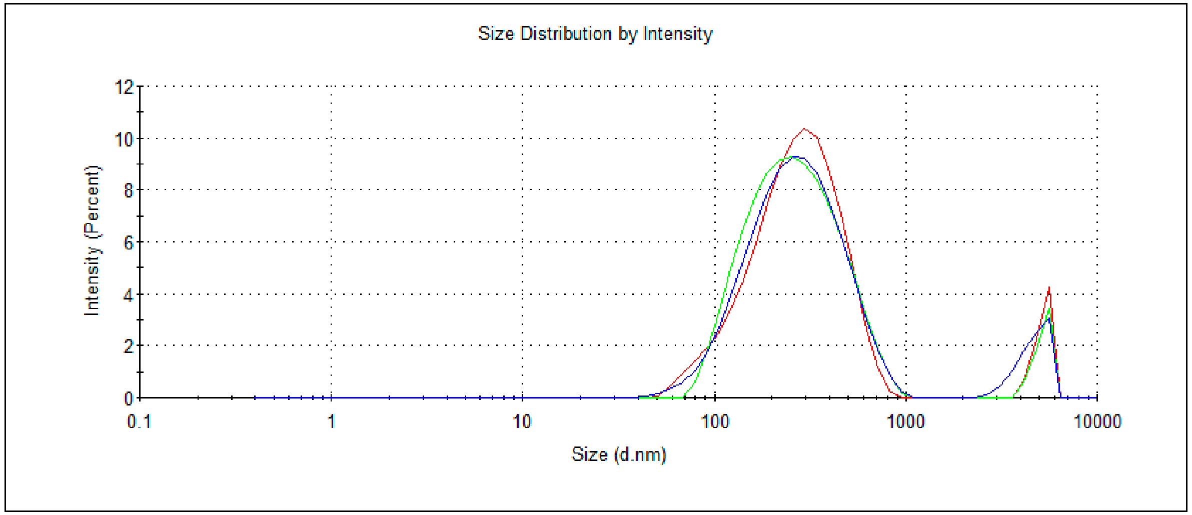

Figure S10. Size distribution of particles by intensity for macrocycle 2 ($1{\times}10^{-3}$ M) in DMSO.

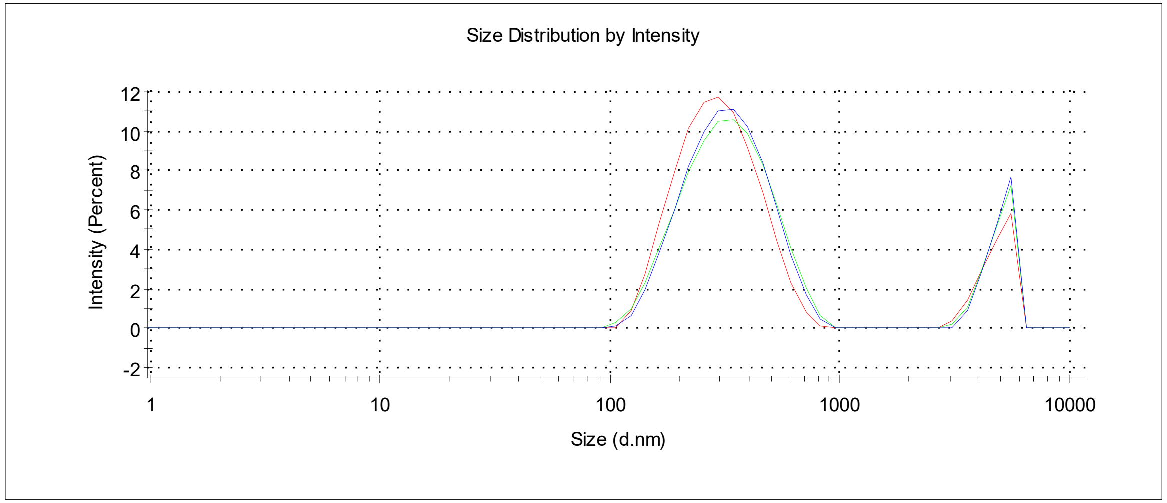

Figure S11. Size distribution of particles by intensity for macrocycle 3 (1×10^{-4} M) in DMSO.

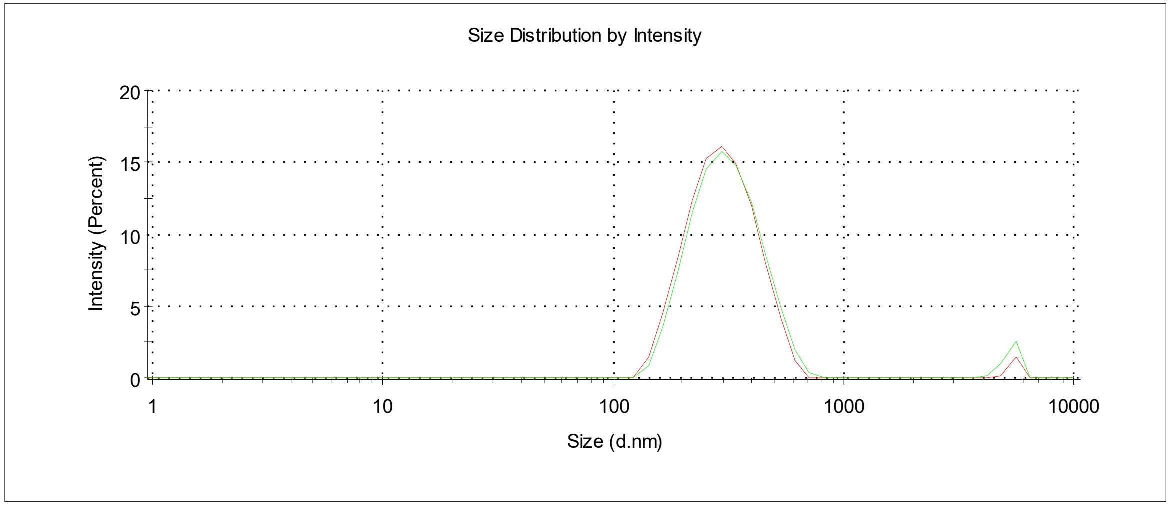

Figure S12. Size distribution of particles by intensity for macrocycle 3 (1×10^{-3} M) in DMSO.

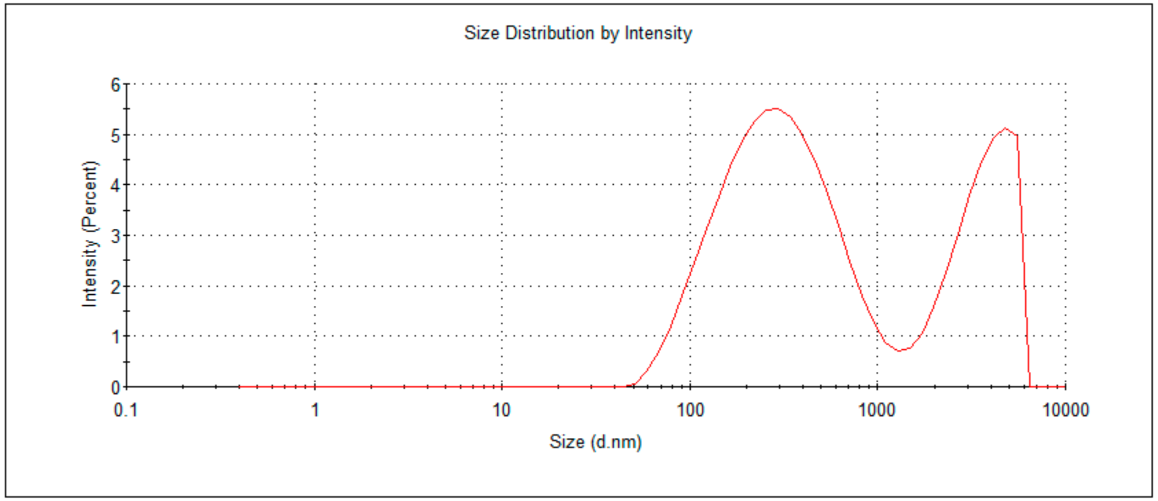

Figure S13. Size distribution of particles by intensity for macrocycle 2 (1×10^{-4} M) in $CHCl_3$.

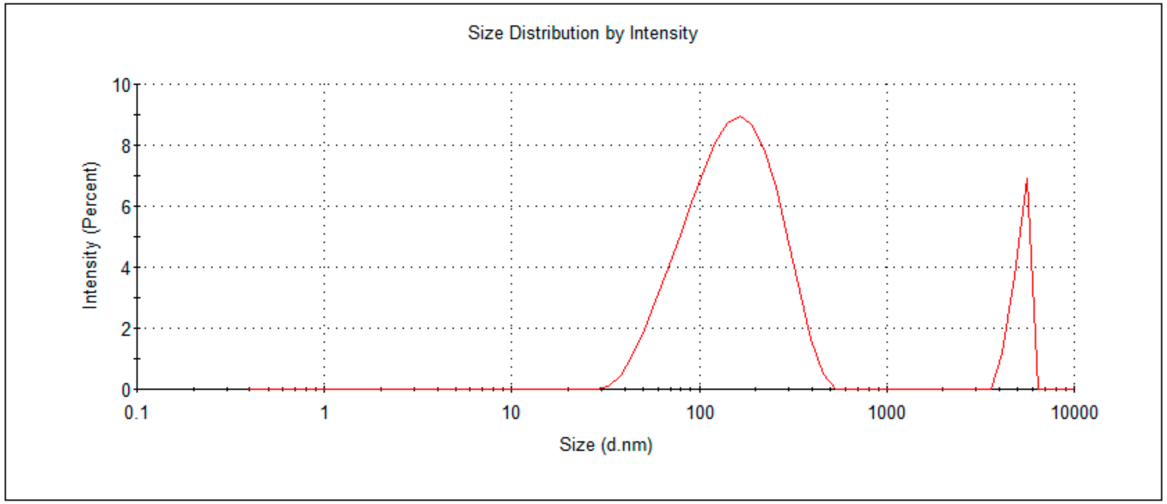

Figure S14. Size distribution of particles by intensity for macrocycle 2 (1×10^{-3} M) in $CHCl_3$.

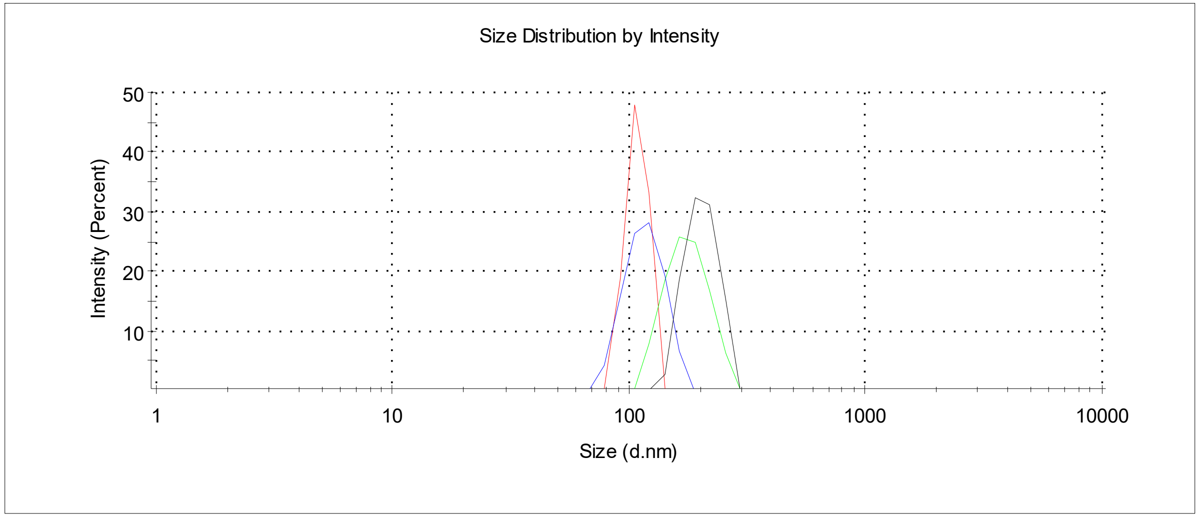

Figure S15. Size distribution of particles by intensity for macrocycle 3 (1×10^{-4} M) in $CHCl_3$.

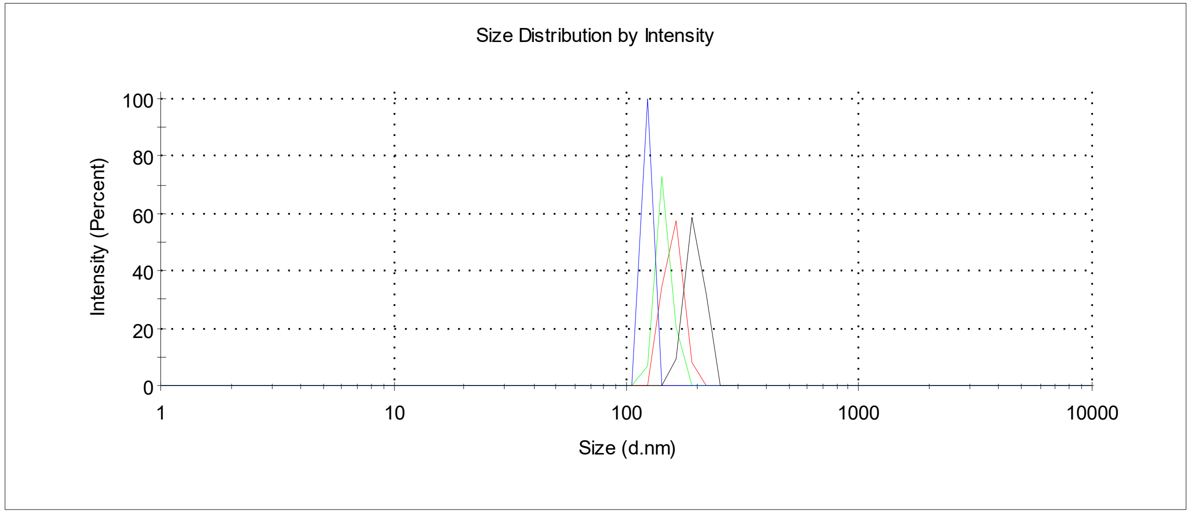

Figure S16. Size distribution of particles by intensity for macrocycle 3 (1×10^{-3} M) in $CHCl_3$.

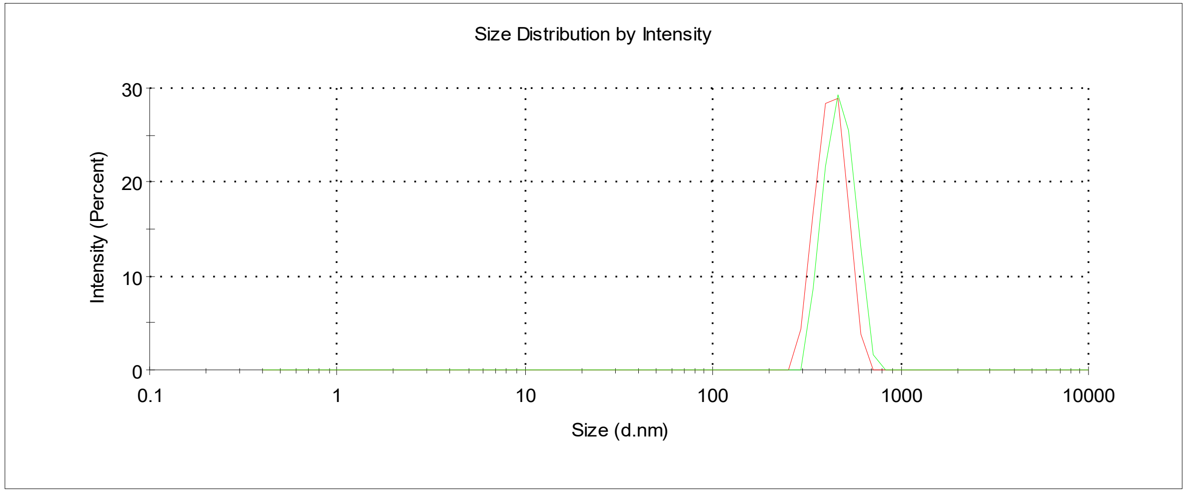

Figure S17. Size distribution of particles by intensity for SLN-2 (3×10^{-4} M) in water.

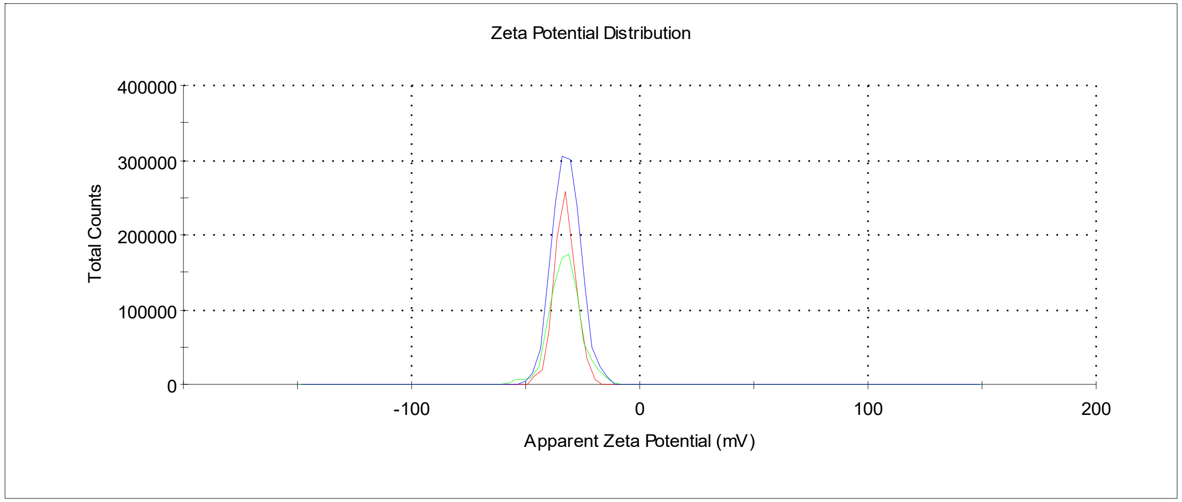

Figure S18. Zeta potential distribution of the particles for SLN-2 (3×10^{-4} M) in water.

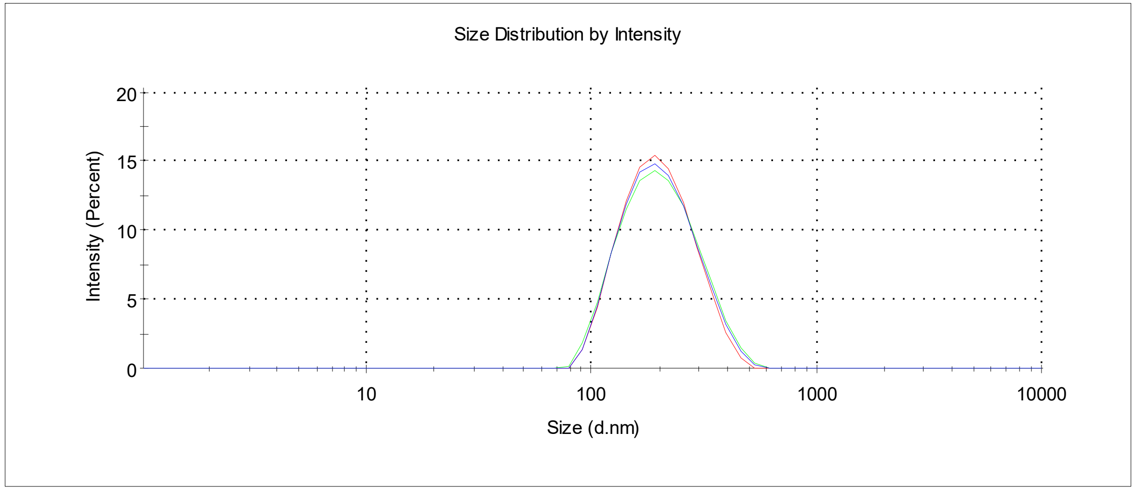

Figure S19. Size distribution of particles by intensity for SLN-3 (3×10^{-4} M) in water.

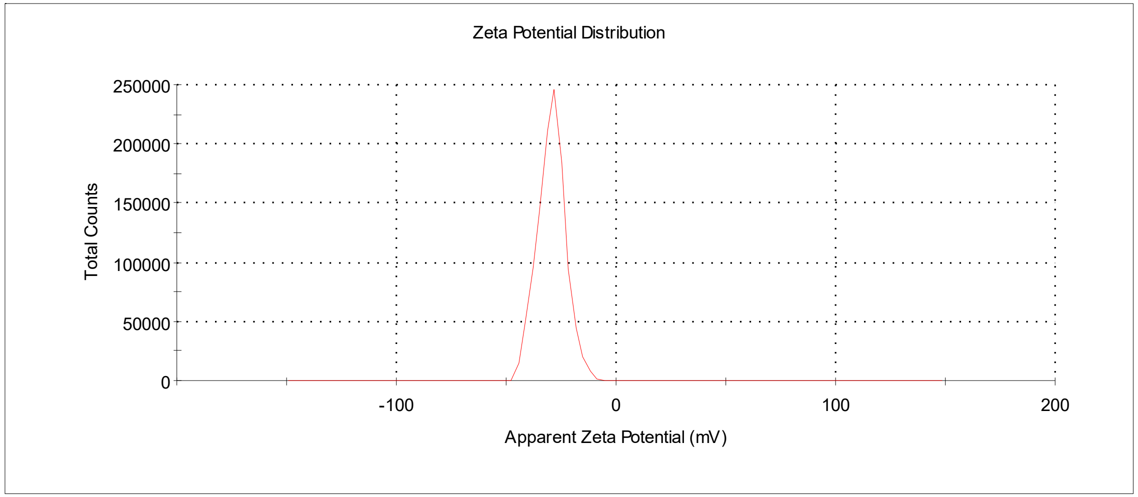

Figure S20. Zeta potential distribution of the particles for SLN-3 (3×10^{-4} M) in water.

References

S1. Yakimova, L.S.; Shurpik, D.N.; Guralnik, E.G.; Evtugyn, V.G.; Osin, Y.N.; Stoikov, I.I. Fluorescein-loaded solid lipid nanoparticles based on monoamine pillar[5]arene: synthesis and interaction with DNA. *ChemNanoMat*. **2018**, *4*, 919-923. doi 10.1002/cnma.201800207
